# Capillary Forces Lead to Pendant Crystals at the Liquid–Air Interface of Evaporating Salt Solutions

**DOI:** 10.1021/acs.langmuir.3c01830

**Published:** 2023-12-05

**Authors:** Simon E. G. Lepinay, Antoine Deblais, Mehdi Habibi, Daniel Bonn, Noushine Shahidzadeh

**Affiliations:** † Institute of Physics, University of Amsterdam, Science Park 904, 1098 XH, Amsterdam, Netherlands; ‡ Department of Agrotechnology and Food Sciences, Wageningen University and Research, Droevendaalsesteeg 4, 6708 PB Wageningen, Netherlands

## Abstract

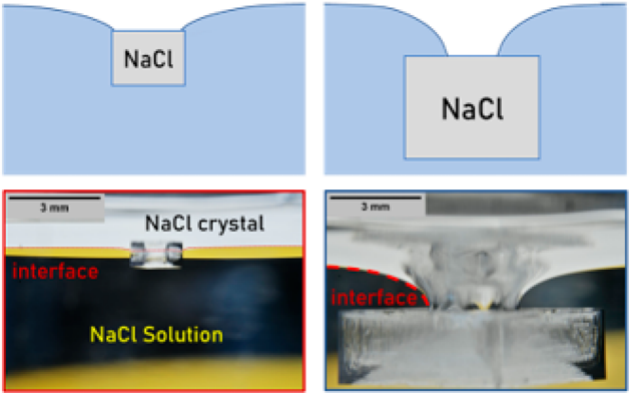

We investigated the nucleation and growth processes of individual NaCl crystals from an evaporating salt solution that is supersaturated. We find that crystals nucleate at the liquid/vapor interface, resulting in distinct “pendant” crystals, which reach millimeter dimensions. The substantial size of the crystals induces deformation of the interface. This process and the evaporation rate, in turn, determine the final crystal shape, which features a deep central cavity. Our findings reveal that a delicate balance exists between gravity, buoyancy, and the surface tension of the liquid/vapor interface that allows the crystal to remain pendant. When the contact angle of the crystal with the meniscus reaches 90°, the crystal disconnects from the interface and falls into the solution. We quantitatively predict the critical mass at which this occurs.

## Introduction

The flotation of objects on the surface of water holds significant importance in aquatic life, water treatment,^1^ and separation technology for extracting precious metals from ore.^2^ Recently, it has also gained attention due to the self-organization of structures at the water’s surface caused by curvature of the meniscus, known as the Cheerios effect^3^ for self- assembling microscale and mesoscale particles at liquid interfaces,^4−6^ with the aim of making materials with different optical and electronical properties.^7^ Generally, small or lightweight objects can float either due to buoyancy or because the surface tension of water prevents excessive surface deformations.^8^ Experiments focusing on capillary-driven self-assembly have predominantly concentrated on the spontaneous formation of intricate patterns utilizing small chemically treated plates that float at a liquid-fluid interface.^9^ Given the small size of these objects, gravity is typically insignificant and is often neglected. In the latter scenario, an additional prerequisite is that the objects possess sufficient hydrophobicity to maintain their stability at the air–liquid interface, which serves as the fundamental principle behind ore flotation.^10,11^ In spite of its importance for a number of applications, several aspects of the problem remain poorly understood. These include determining the size and density of objects capable of floating at an interface and their relationship with the interfacial properties. Furthermore, it is essential to explore how particles such as crystals evolve and grow in size compared to crystal growth phenomena. Whether a precipitating crystal forms in the bulk or at the surface has been shown to depend on the wetting properties of the emerging salt crystals for the liquid–vapor interface.^12^ Recently it was also shown that the preferential arrangement of anions and cations near the surface^13^ modifies the free energy for crystal nucleation from solution.^14^

Despite sporadic photographic observations of floating salts in experiments as well as in locations such as the Dead Sea and salt mines, this phenomenon has predominantly received little attention. Few studies have been dedicated to investigating the formation of “fleur de sel” (Flower of Salt), characterized by hollow pyramidal crystals that float and grow on the surface of ponds during hot summer days. Notably, these occurrences have been reported in coastal areas of Brittany (France) and the Algarve region of Portugal.^15,16^

In this study, we investigate the floating behavior of growing salt crystals of Halite (NaCl) that form at the free surface of saturated salt solution during evaporation similarly to the study of Davies et al. (2021)^17^ who studied the interactions between a solid cube under gravity and a horizontal soap film. NaCl crystals generally can grow by three morphologies dictated by its FCC lattice and denoted with Miller planes as cube {100}; octahedron {111}; and rhomb-dodecahedron {110} with a strong tendency to form cubic crystals {100}.^18^ The surface energies of different crystallographic facets can vary depending on the atomic arrangement and bonding within the crystal lattice. For example, the {100} facet is typically considered to have the lowest surface energy, followed by the {110} and {111} facets.^19^

Here, we report on the first systematic study of the nucleation and growth of NaCl face-centered cubic growth (FCC) of {100} crystals at the interface from solution. These nucleate preferentially at liquid/air, and we study their growth until they reach a macroscopic size while floating. We show that although the NaCl crystals have a significantly larger density than the surrounding salt solution, they can float and continue to grow while deforming the interface until the capillary forces are no longer strong enough to maintain the weight of the macroscopic crystal. The crystal then falls to the bottom of the vial, and the nucleation continues with the formation of a new crystal(s) at the interface from secondary nucleation. The growth dynamics of the floating crystal at the interface is described by 2 different regimes, before and after the bending of the liquid/vapor interface. The latter results in the formation of a central cavity in the cubic crystal during the layer by layer growth of the side and bottom crystal faces submerged in the salt solution. We also show that the equilibrium between the weight of the crystal and the bending of the surface gives a similar force balance as the classical equilibrium in the pendant drop system which is frequently used for the determination of surface tensions.^20^ Therefore, by analogy, the floating NaCl crystal allows for the determination of the surface tension of the liquid at the vicinity of a growing crystal. Finally our results also call into question the general assumption that a salt crystal is generally wettable by its own salt solution.

## Experimental Section

### Solution Preparation

To conduct the bulk evaporation experiments, homogeneous and slightly undersaturated solutions of NaCl (BioXtra, >99.5%) with a concentration of approximately 5.9 mol/kg were prepared (the saturation being at 6.1 mol/kg). For each experiment, a mother solution was created by dissolving 34.9 g of NaCl in 100 mL of water (Millipore, ρ ≈ 18.2 MΩ cm) at a temperature of *T* = 21 °C. The solutions were thoroughly stirred for a period of 6 h to ensure complete dissolution and homogeneity throughout the solution.

### Evaporation Setup

**Figure 1:**
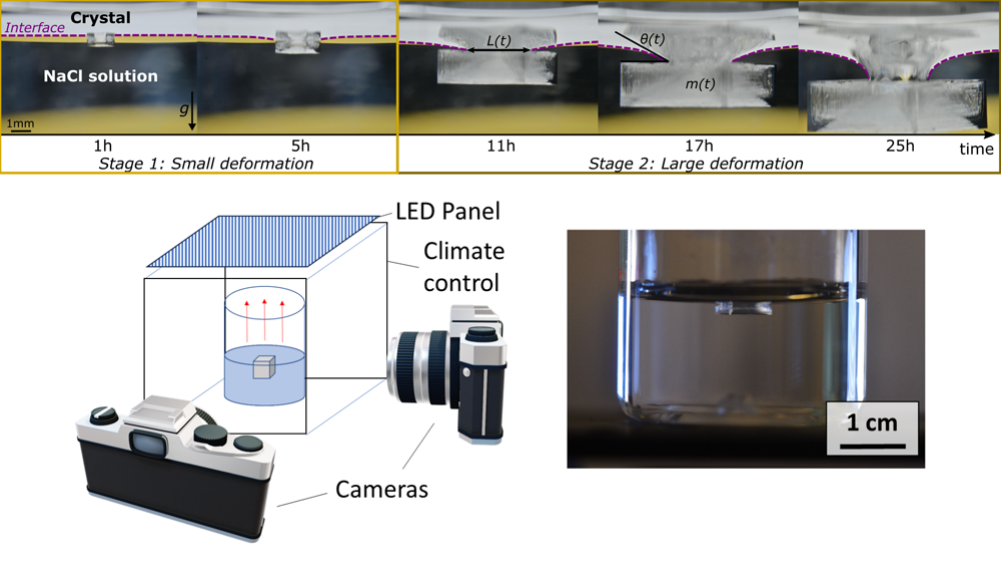
(Top) Sequence of images depicting the typical nucleation of a NaCl crystal at a salt solution/air interface (purple dashed line) during evaporation (black and yellow background are the bulk salt solution region) under controlled atmospheric conditions (RH = 50% and *T* = 21 °C). The crystal growth occurs in two distinct stages characterized by differing levels of deformation of the interface. In stage 1, the interface exhibits minimal deformation as the crystal grows, while in stage 2, a pronounced deformation is observed along with a shift in the contact line. Contact line length *L* (t), contact angle θ (t), and crystal mass *m* as a function of time *t* are explained in the text. (Bottom, left) Schematic of the setup. (Bottom, right) Side view (wide) of the crystal nucleating at the air–liquid interface.

For the evaporation process, glass containers were made by cutting the top sections of vials. Each recipient was filled with 10 g of aqueous salt solution and left to dry in a controlled environment. Throughout the experiments, both the relative humidity (RH) and temperature (*T*) were carefully monitored. The RH was kept at 50 ± 2.5% in a controlled climatic chamber, while T was kept at 21^◦^C. These controlled conditions ensured a moderate evapo-ration rate with the nucleation of single crystal at the free surface in most of the cases. The salt concentration is fixed by the solubility and in part by the evaporation rate. In this experiment, we tried several different relative humidities, but only a sweet spot at 50% ± 5% allowed for consistent single crystal nucleation at the surface. To minimize the likelihood of multiple nucleation events further, the top surface of the cylindrical bottle was covered with micrometer size filters to prevent dust contamination. Additionally, the entire setup was positioned on an antivibration optical table to further reduce any external disturbances. Regular interval photographs were taken during the evaporation process using several conventional photo cameras (Canon D5300) with macro-objective lenses (Sigma). Throughout each experiment, one camera was focused on the free surface of salt solution–air, whereas the second camera was capturing the general view of the whole bottle. For optimal temporal resolution, the frame-rate of the camera was set at 50 frames/s after nucleation. The typical duration of a single evaporation experiment was approximately 3 days. Due to the experiments’ time scale and their sensitivity to perturbations and considering that crystallization is a stochastic process, only experiments in which a single crystal nucleated and grew at the free surface were considered for this study. In total, we conducted over 100 experiments. Out of these 100 experiments, ∼20% resulted in the formation of single crystals with their {100} facet oriented along the surface (as seen in Figure 1). The preferential nucleation of crystal at the liquid–vapor interface is robust, and has been previously studied in the context of drying sessile droplet.^12,21^ The remaining experiments were unsuitable for study due to a combination of crystal growth along the 111 direction, the occurrence of multiple nucleation events at the surface, and nucleations at the bottom of the container.

### Crystal Characterization

After nucleation, the floating crystals at the free surface were collected at different stages of growth in order to analyze their size and shape. In addition, in some other experiments the growth was followed until the final stage of floating: when a macrocrystal becomes too large, it detaches from the interface and falls to the bottom of the recipient. The crystals were then promptly removed from the solution and rinsed with pure ethanol (purity >99%). This step effectively eliminated any remaining traces of salt solution from the crystal surfaces. The crystal sizes and shapes were then assessed using a Hitachi TM3000 scanning electron microscope (SEM) and a Keyence-VX100 3D laser profilometer with a wavelength of 404 nm. Furthermore, volume measurements were done by submerging the crystals in ethanol and recording the corresponding rise in surface level. Knowing the mass and the volume of the crystal the apparent bulk density was determined for each crystal.

## Results and Discussion

As shown in Figure 1, we typically observed the nucleation of a NaCl crystal, followed by its further growth, and eventually its fall into the solution as its weight overcomes the capillary forces, keeping it afloat. Our experimental findings reveal two distinct stages during the crystal’s growth:

(i) In the first stage, which begins right after nucleation, the crystal’s edge acts as a pinning point for the liquid/air interface. Due to the small mass of the nascent crystal, the solution surface undergoes minimal deformation (1 and 5 h in Figure 1).
(ii) In the second stage, the growth and weight increase of the crystal causes a large deformation of the liquid/air interface. The curved interface at a certain stage in the growth remains pinned near the center of the top facet.

**Figure 2:**
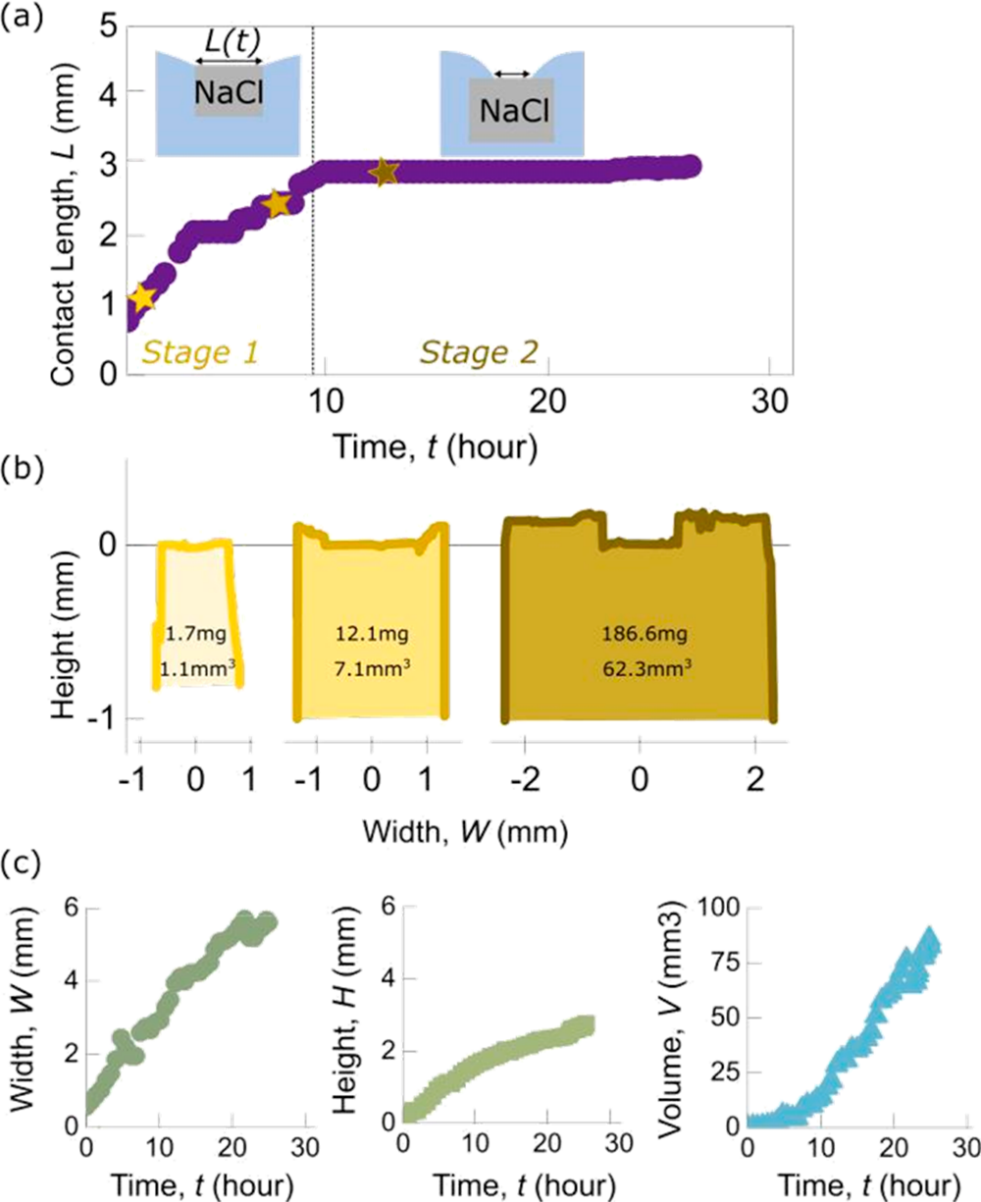
(a) Evolution of the length *L*(*t*) of the contact line between the free surface and the crystal during the growth of a pendant crystal. (b) Vertical cross sections of a single crystal obtained using 3D laser profilometry at the three time points indicated by stars in (a). Heights are aligned with the bottom of the cavity and their width centered at 0. (c) Growth rates of the crystal’s height, width, and volume.

To differentiate between these two stages, we analyzed the length *L*(*t*) of the contact line between the fluid meniscus and the crystal. In stage 1, as the crystal grows, the contact length increases with increasing crystal size (Figure 2a). At the onset of stage 2, surprisingly, the contact line reaches a plateau, indicating that it becomes pinned at a specific size while the crystal continues to grow.

**Figure 3:**
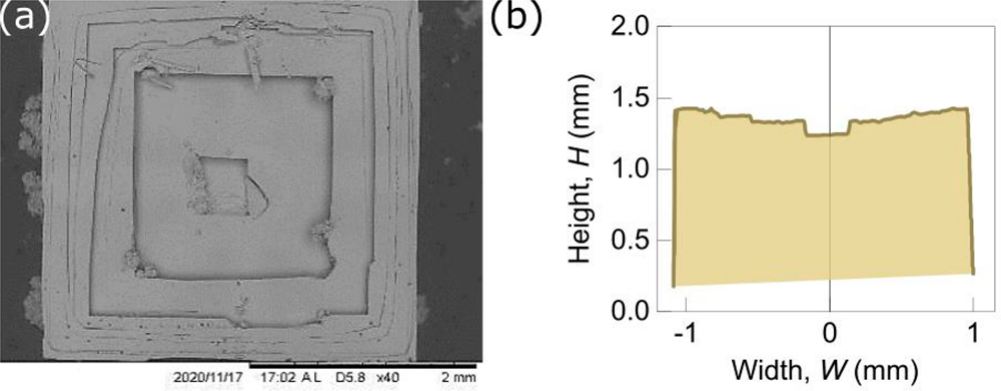
(a) SEM top-view picture of a typical crystal cavity. (b) Cavity profiles obtained from confocal profilometry for a crystal collected at the final stage 2 of growth.

The analysis of the crystals at different stages of growth shows that in stage 2, when the crystals exceed a mass of about 10 mg, they exhibit a cavity with sharp edges; cross sections of crystals’ top facets measured at a late stage of growth by laser profilometry are shown in Figure 3b.

**Figure 4:**
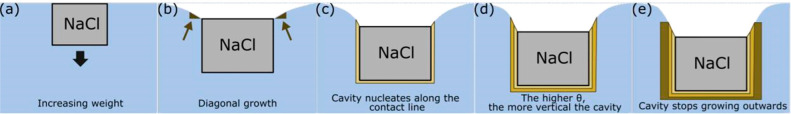
Mechanism of cavity formation during pendant crystal growth. In the initial stages (a), the pendant crystal has a small weight, allowing it to remain attached to the liquid/air interface. As the crystal continues to grow, the contact line undergoes deformation (b), leading to diagonal growth along the interface (c). The vertical extent of the cavity increases as the crystal grows heavier, resulting in a higher contact angle θ (d). Eventually, the cavity reaches a limiting size (e).

The transition between the two regimes and the formation of a cavity can be explained as follows. Immediately after nucleation at the free surface of the salt solution, the crystal is so small that its weight has a negligible effect on surface deformation (Figure 4a). Only facets exposed to the solution are capable of growth, allowing for horizontal and downward growth only. As the crystal continues to grow, its increasing weight starts to deform the salt solution/air interface. It has been demonstrated that particles with sharp edges are more likely to float compared to particles with smooth surfaces;^22^ for our case of cubic crystals, the capillary attachment force is greater than that of a spherical particle with the same wet perimeter and contact angle^2,22^ (Figure 4a). The pinned bent meniscus then acts as a reservoir of salt solution for the upper edges of the crystals, enabling horizontal growth (diagonally) in these regions as well (Figure 4b). Consequently, the crystal continues to grow upward, sideways, and downward as water evaporates (Figure 4c–e). With the deposition of new layers of salt on the crystal surfaces, the crystal expands in size, while the contact line remains fixed to the original edge. The gradual increase of the contact angle of the meniscus, caused by the bending due to the increased weight of the crystal, allows for perfectly vertical growth, ultimately resulting in the formation of the cavity until the contact angle approaches 90°. While the weight of the crystal increases continuously, the three-phase contact line makes discontinuous jumps as a consequence of the stochastic nature of the contact line anchoring and hysteresis, resulting in the observed steps (Figure 3a). The steps correspond to a discontinuous motion of the three-phase contact line between the crystal, the fluid, and the vapor.

**Figure 5:**
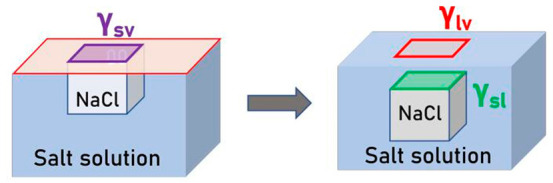
Schematic representation showing the interfaces involved in the crystal’s detachment from the liquid/air interface.

To examine the growth process in more detail, we write the condition for the crystal’s flotation in terms of interfacial tensions; as illustrated in Figure 5, the system has the lowest surface energy for a crystal forming at the liquid/air interface when
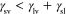
1where subscripts l = liquid, v = vapor, and s = solid. For salt solutions, the liquid/vapor surface tension is higher than pure water and increases with the concentration of ions in the solution; for a saturated NaCl solution (6.1 mol/kg), we measured γ_lv_ ≈ 81 mN/m using the pendant drop method. The crystal/vapor surface tension is notoriously difficult to measure, and literature values differ by almost an order of magnitude.^23^ To nonetheless obtain an estimate, we use the approximation proposed by Israelachvili:^24^
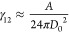
2where γ_12_ is the interfacial tension between the media 1 and 2 (in mN/m), *D*_0_ is the intermolecular cutoff (0.165 nm), and *A* is the Hamaker constant:
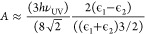
3where ϵ_1_ denotes the dielectric constant of the solid (80), the solution ϵ_2_ = 1, the vapor ϵ_2_ = 4.9, the Planck constant *h* = 6.63 × 10^–34^J s, and the plasma frequency of the free electron gas ν_UV_ = 5 × 10^–13^/s. We find γ_sv_ ≈ 30.9 mN/m and γ_sl_ ≈ 4.5 mN/m by considering media 1 to be the NaCl crystal phase (solid) and media 2 is either the liquid (salt solution) or vapor phase (air). These estimates underlying the lower surface tension of the solid phase agree with eq 1, explaining why the crystal forms at the liquid/air interface. This could, in principle, be due to the fact that the salt concentration is higher there because the evaporation concentrates the ions near the interface. However, we do not anticipate significant heterogeneity in the solution as the evaporation at *T* = 21 °C, RH = 50% is slow (several days for 10 mL of solution), so the Peclet number will be very low as well. In addition, the corresponding experiment with an evaporating droplet of NaCl solution^21^ also shows that even if the concentration gradients are stronger at the contact line,^25^ crystals form near the apex of the drop. For cubic crystals such as the ones formed here, the 6 facets are equivalent, and since these crystals form at the solution–air interface, the Young equation tells us that the solution does not wet the crystal-air interface. In agreement with this, measurement of the contact angle that a saturated salt solution makes on a high quality single NaCl crystal (IR window) gives a contact angle of 8 ± 2° (see Figure S1). This confirms again that the salt solution does not fully wet the surface.

**Figure 6:**
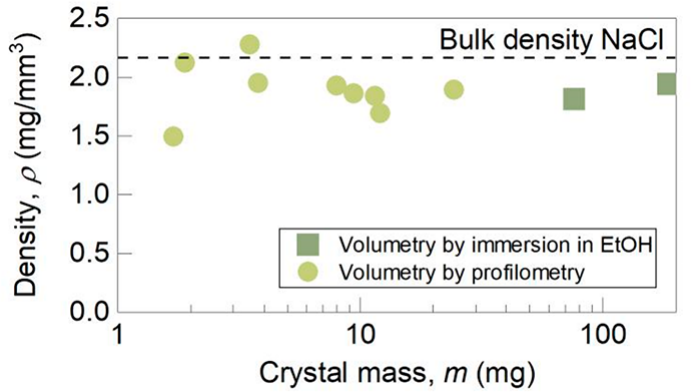
Pendant crystals density determined from two methods: Profilometry and ethanol submersion; data compared with the theoretical value, 2165 mg/mm^3^ (dotted solid line).

To determine the conditions for crystal flotation, we consider the force balance between gravity, *F*_g_, buoyancy force, *F*_b_, and capillary forces, *F*_cap_, for a crystal of volume *V* and contact perimeter *L*. Here, the capillary force depends only on the surface tension between salt solution and air γ_lv_ because it is the only interface that can deform.
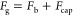
4where the capillary force *F*_cap_ can be written as the surface tension times the perimeter length of the contact line *L*, leading to the force balance in the *z*-direction:
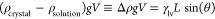
5so that sin(θ) ∼ *V*Δρ*g*/γ_lv_*L*. The density of the crystal has been obtained from two different experimental techniques (Figure 6); profilometry allows us to get an accurate volume for the smaller crystal’s size, while the immersion method (ethanol) allow us to determine density for larger crystal mass (*m* > 50 mg).

**Figure 7:**
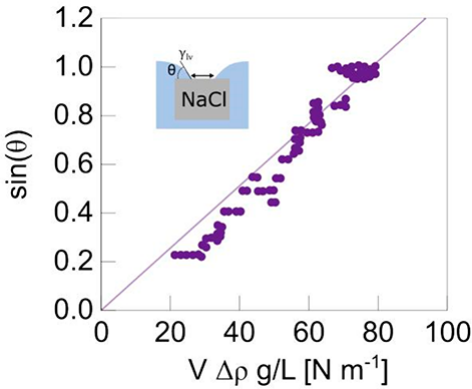
Sine of contact angle θ as a function of *V*Δρ g/L for a pendant crystal in stage 2 with the deformed interface pinned at the edges of the cavity. The solid line is a linear fit.

We plot sin(θ) against *V*Δρ*g*/*L* in Figure 7. From a linear fit, we find that γ_lv_ = 79 ± 3 mN/m, which is consistent with the value of 81.5 mN/m for saturated salt solution.^25^ The force has a component parallel and perpendicular to the interface; the latter increases when the angle becomes larger and is maximum at an angle of 90°. This, in turn, determines the maximum weight of the crystal that can remain suspended. From this we can also deduce the maximal mass for a crystal attached to the surface from the maximum capillary upward force; we find ∼300 mg, consistent with the maximum weight of the crystal (310 mg) measured experimentally after sinking in our series of experiments. In the hypothesis of a full cubic crystal, we can substitute the contact line length with the full perimeter of the crystal in eq 5, and in the limit where sin(θ) = 1, the maximum weight more than doubles.

From this data, we can compute a critical Bond number, *B*_0,c_:
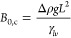
6that we found in our case *B*_0,c_ ∼ 0.5, consistent with values reported from simulation and experiment for a cubic crystal.^17^ Density and shape of objects floating at an interface are crucial ingredients that determine the critical number at which they will detach from the interface.^8^ Here, in this situation, the nucleation process gives rise to a specific {100} crystal; this shape could be changed through nucleation of other type of salt (e.g., calcite or potassium alum) at the air/liquid interface, that may lead to another type of crystal shape such as rhombi^26^ or hexagonal.^27^ For such a shape, one could check if the critical Bond number at which the crystal falls is universal.

**Figure 8:**
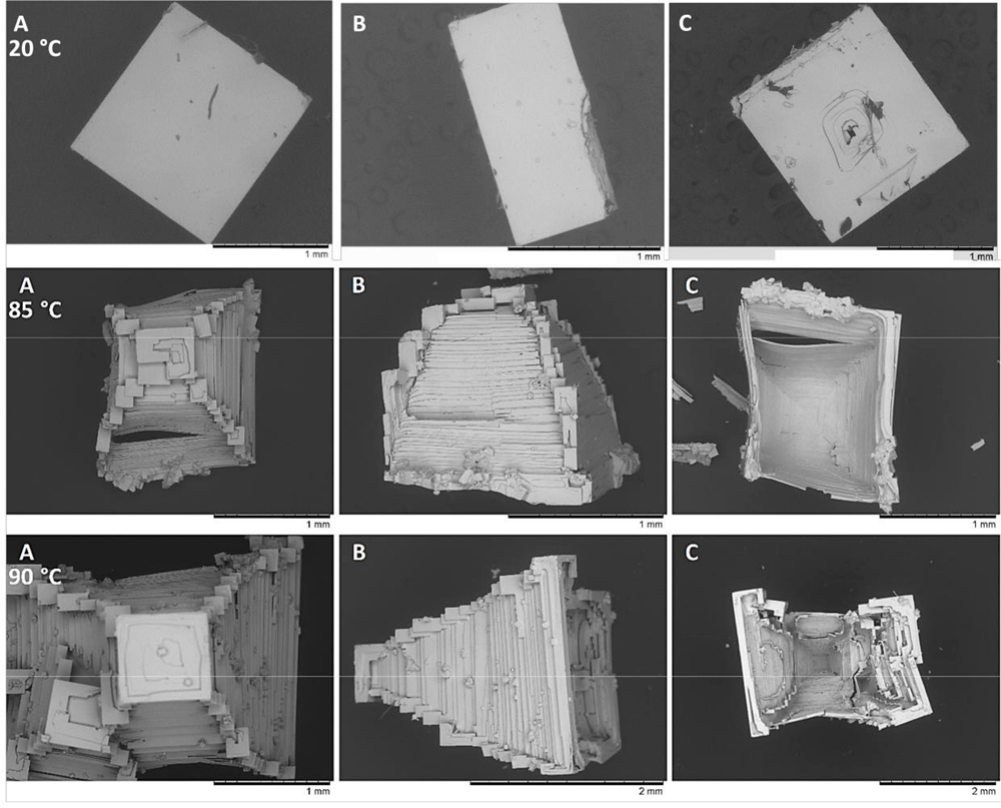
SEM pictures of single NaCl crystals grown at the solution–air interface for three different temperatures (top to bottom: 20 °C, 85 °C, and 90 °C). The crystals are collected from the evaporation of 50 mL of an initial salt solution at 5 mol/kg. A, B, and C are, respectively, the bottom, side, and top facets of the crystals.

Finally, it should be noted that the evaporation rate is an important factor determining the geometry of the cavity during the growth of the crystal at the liquid/vapor interface. Recently, it has been shown that high evaporation rate can increase the total volume of inclusions in growing crystals in a solution,^28^ affecting their density. In the case of pendant crystals, a series of experiments done at different temperatures up to 90 °C show clearly that the higher the temperature, the deeper the cavity becomes (Figure 8). Although high evaporation rates lead to high nucleation rates, making the observation of single pendant crystals scarce. In our experiment, the measured evaporation rate at 90 °C (0.03 mm/s) allows for the evaluation of Peclet number, Pe, designating the relative importance of ion advection and ion diffusion.
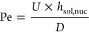
7Here, *U* is the characteristic fluid velocity (advection rate) or the evaporation rate of the solution. In our case, we estimated it from the change of height of the liquid in time d*h*_sol_/d*t* (see Supporting Information, Figure S2); *h*_sol,nuc_ is the characteristic length or characteristic dimension (e.g., the size of the system or the distance over which the substance is transported); *D* is the diffusion coefficient of the NaCl solution, *D* = 1.6 × 10^–5^ cm^2^/s)). Taking the characteristic length scale of the Peclet number to be the fluid depth (in the beaker) at the moment the first crystals are observed, we find *P* ∼ 500. This is very large, much larger than unity, showing that ions are strongly advector to the evaporative surface. This in turn leads to a much faster growth of the crystal at its edges close to the liquid–vapor surface compared with the bulk growth of the submerged faces. This anisotropic growth results in the creation of larger and deeper cavities with a pyramidal morphology.

## Conclusions

We studied the evaporation of NaCl salt solutions in a hydrophilic glass recipient and found that NaCl crystals nucleate systematically at the liquid/air interface and continue to grow up to millimeter-sized while remaining suspended at the interface. The growth proceeds in two distinct stages. First, the crystal facets in contact with salt solution grow sideways layer by layer without affecting the liquid/air interface. Second, as it grows and becomes heavier, it pulls down the liquid/air interface, which will facilitate the growth of the top surface at the edges, leading to cavity formation in the cubic layered crystal. The cavity forms because when the contact line remains anchored at the surface of the crystal, the part of the surface that is in contact with the solution continues to grow but the part that is in contact with the vapor not. Such growth results in a cubic crystal with a slightly lower density compared to that of the same type of cubic crystal grown from a bulk solution. Finally, when the bending angle of the meniscus reaches 90°, the crystal completely sinks into the salt solution, leaving room for another crystal to possibly form.

By considering surface and interface energy balances, we show why the crystal initially remains suspended, although its density is higher than the salt solution. The contact angle is directly related to crystal and solution parameters, revealing a critical mass below which the crystal remains floating as it grows. The continuous growth on curved liquid/air interface is also in good agreement with previous studies showing that NaCl crystallization can take easily the shape of curved liquid capillary bridges in confinement.^29^ It may therefore also be feasible to obtain the controlled growth of certain crystal shapes or size by tuning the surface properties, e.g., by using surfactants or evaporation rate.^25,30^ In addition, the results could also explain the pyramidal growth of “fleur de sel”, which occurs at higher temperature; higher evaporation rate induces a larger advection of ions at the evaporative surface and induces a faster growth of the top facet, leading to the creation of larger cavities and subsequently a pyramidal morphology.

Studying crystal growth at the free surface offers valuable insights into the influence of interfacial phenomena on the kinetics and mechanisms that govern crystal nucleation and growth. By carefully manipulating growth conditions, such as the temperature and evaporation rate, it becomes possible to exert control over the shape, size, and surface characteristics of the crystals. This level of control is particularly significant in various applications in which specific crystal morphologies are desired for their distinct properties or functionality. Conversely, the precise details of crystal shape and their point of formation hold paramount importance in understanding the damage caused by salt crystallization in porous materials, such as building materials and stone artworks.^31^

## Supporting Information Available

Additional details on the contact angle measurement of a saturated NaCl solution at the surface of a NaCl crystal and on the evaporation rate of the solution (PDF)
