# Supplementary material for: Capillary Forces Lead to Pendant Crystals at the Liquid–Air Interface of Evaporating Salt Solutions
Source: Langmuir. 2023 Dec 5;39(50):18208–14. doi: 10.1021/acs.langmuir.3c01830 (PMC10734214; doi:10.1021/acs.langmuir.3c01830)
Supplement: Supplementary file 1 — la3c01830_si_001.pdf [file la3c01830_si_001.pdf]

Supplemental Material for:
Capillary Forces Lead to Pendant Crystals at the Liquid-Air Interface of Evaporating Salt Solutions

Simon E. G. Lepinay,[1] Antoine Deblais,[1] Mehdi Habibi,[2] Daniel Bonn,[1] and Noushine Shahidzadeh[1, a)]

[1)] *Institute of Physics,*
University of Amsterdam,
Science Park 904,
1098 XH,
Amsterdam,
Netherlands

[2)] *Department of Agrotechnology and Food Sciences,*
Wageningen University & Research,
Droevendaalsesteeg 4,
6708 PB Wageningen,
Netherlands

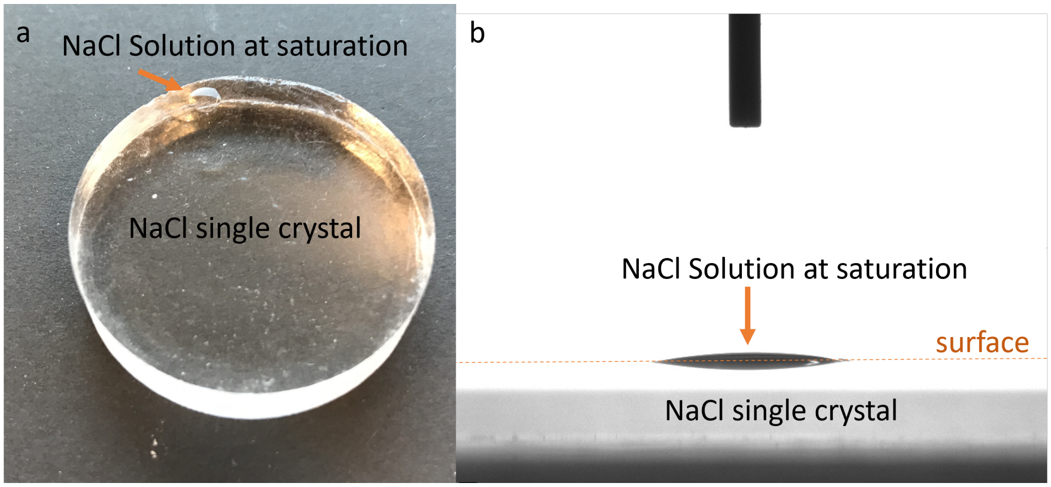

FIG. S1: Contact angle measurement of saturated NaCl solution at the surface of high-purity NaCl crystal (cell windows). (a) top view ; (b) side view using a Krűss scientific instrument for contact angle measurements. The average contact angle is: θ=8±2°.

a) Electronic mail: N.Shahidzadeh@uva.nl

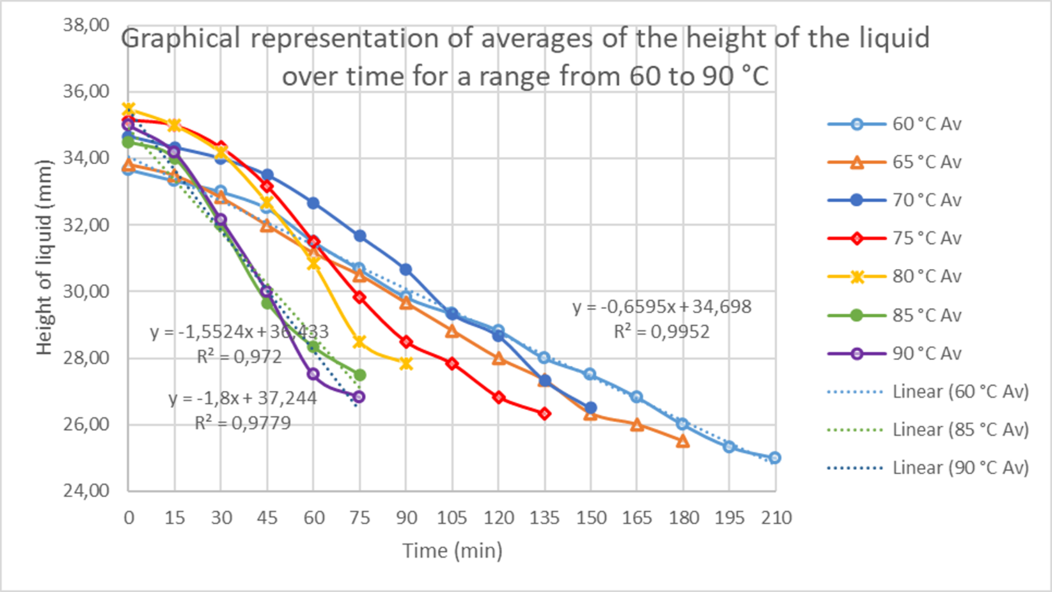

FIG. S2: Average liquid height, h_{sol} variation over time for the evaporation of 5 mol/kg NaCl solutions at constant temperature
